# The safety and short-term efficacy of nivolumab plus ipilimumab for advanced esophageal squamous cell carcinoma

**DOI:** 10.1007/s10388-025-01162-3

**Published:** 2025-10-26

**Authors:** Kazuhiro Shiraishi, Shun Yamamoto, Yuri Yoshinami, Nozomu Ogura, Mai Itoyama, Hiroshi Imazeki, Kazuki Yokoyama, Yoshitaka Honma, Tairo Kashihara, Daisuke Kurita, Koshiro Ishiyama, Junya Oguma, Hiroshi Igaki, Hiroyuki Daiko, Yasuyuki Seto, Ken Kato

**Affiliations:** 1 Department of Head and Neck, Esophageal Medical Oncology, National Cancer Center Hospital, 5-1-1 Tsukiji, Chuo-Ku, Tokyo, 104-0045 Japan; 2 Department of Radiation Oncology, National Cancer Center Hospital, Tokyo, Japan; 3 Department of Esophageal Surgery, National Cancer Center Hospital, Tokyo, Japan

**Keywords:** Esophageal squamous cell carcinoma, Nivolumab, Ipilimumab, Immune-related adverse events

## Abstract

**Backgrounds:**

Nivolumab plus ipilimumab (Nivo + Ipi) is recommended as the first-line standard treatment for patients with advanced esophageal squamous cell carcinoma (aESCC), taking into consideration the patient’s general condition and Programmed cell death ligand 1 (PD-L1) expression. In the CheckMate 648 (CM648) trial, the incidence of treatment-related adverse events (TRAEs) of any grade and grade ≥ 3 were reported to be 80% and 32%, respectively, among patients who received Nivo + Ipi Still, it is occasionally given to patients with frail general conditions or as salvage-line treatment. However, there was little data on safety and efficacy in real-world settings.

**Methods:**

We retrospectively analyzed the data of patients who received Nivo + Ipi for aESCC between 2022 and 2023 in our hospital. We evaluated immune-related AEs (irAEs), overall response rate (ORR), progression-free survival (PFS), and overall survival (OS).

**Results:**

Thirty patients were subjected to this study. The patients’ characteristics were as follows: median age (range): 63.5 (36–80) years, ECOG PS 0/1 ≤ : 16/14, treatment-line 1st/2nd or later: 6/24. Eight of 30 patients who experienced grade 2 or higher irAE required systemic steroid therapy. Four of 8 patients required additional treatment following the initial steroid therapy, two with increased steroid dose, one with mycophenolate mofetil (MMF), and one with steroid pulse therapy plus MMF. The ORR, median PFS, and median OS were 66.7%, 11.0 months, and 15.4 months in the 1st-line group and 36.8%, 2.6 months, and 10.2 months in the 2nd or later-line group, respectively.

**Conclusions:**

Our study showed a safety profile comparable to that of CM648 trial. Nivo + Ipi as 2nd or later-line treatment demonstrated promising efficacy.

## Introduction

Esophageal cancer (EC) is the sixth leading cause of cancer deaths in the world [1], and EC is divided into two major subtypes. One is squamous cell carcinoma, the most common in Asian countries, and the other is adenocarcinoma, which is common in Western countries [2]. Although there has been no evidence of systemic chemotherapy which prolonged survival compared with the best supportive care for advanced esophageal squamous cell carcinoma (aESCC), cisplatin plus 5-fluorouracil (CF) therapy had been recognized as the 1st-line standard treatment [3, 4].

In the CheckMate 648 (CM648) trial, which was a phase III trial comparing nivolumab (Nivo) plus CF or Nivo plus ipilimumab (Nivo + Ipi) to CF in the 1st-line setting advanced ESCC patients, programmed cell death ligand-1 (PD-L1) expression was evaluated using the tumor proportion score (TPS), defined as the ratio of PD-L1 positive tumor cells divided by the total number of tumor cells multiplied by 100. The median overall survival (OS) was improved significantly with Nivo + Ipi than with CF in both TPS ≥ 1% population [13.7 vs. 9.1 months, hazard ratio: HR (98.6%confidence interval: CI); 0.64 (0.46–0.90)] and in the overall population [12.7 vs. 10.7 months, HR 98.2%CI; 0.78 (0.62–0.98)]. The incidence of any grade and grade ≥ 3 treatment-related adverse events (TRAEs) by Nivo + Ipi were 80% and 32%, respectively [5]. However, the irAEs frequency of Nivo + Ipi in real-world settings remains unknown.

In addition, the neoadjuvant triplet regimen [docetaxel, cisplatin, and 5-fluorouracil (DCF)] of resectable ESCC has been the standard of care in Japan [6]. If the disease progressed within six months after neoadjuvant DCF, either Nivo alone or combination of Nivo + Ipi is used as systemic therapy. However, there are few reports of safety and efficacy of Nivo + Ipi in patients who have failed platinum- containing regimens.

## Materials and methods

### Patients

We retrospectively analyzed the patients with the following conditions: histologically confirmed ESCC, clinical stage IVB (Union for International Cancer Control tumor-node-metastasis classification in 8th edition) or recurrence with no indication of curative treatment, Easter Cooperative Oncology Group Performance Status (ECOG PS) 0–2, with evaluable lesion, and treated with Nivo + Ipi between 2022 and 2023 at the National Cancer Center Hospital (NCCH). The 1st-line (1L) was defined as no prior systemic therapy for advanced ESCC or recurrence after 6 months since the last dose of chemotherapy on the multidisciplinary approach for locoregional disease. The 2nd or later-line (≥ 2L) was defined as previously treated with systemic therapy for advanced ESCC or recurrence within 6 months since the last dose of chemotherapy on the multidisciplinary approach for locoregional disease. This study was approved by the Institutional Review Board of NCCH (Approval No. 2020–287) and conducted in accordance with the Declaration of Helsinki.

### A regimen of nivolumab plus ipilimumab therapy

Treatment with the Nivo + Ipi regimen consisted of Nivolumab: 360 mg/body repeated every 3 weeks, and Ipilimumab: 1 mg/kg repeated every 6 weeks. Treatment was continued until obvious disease progression, unacceptable toxicity, or the patient’s refusal to continue the treatment.

### Assessments and statistical criteria

The Common Terminology Criteria for Adverse Events (CTCAE Ver. 5.0) was used to evaluate the irAEs. OS was defined as the period from the date of treatment initiation to the date of any cause of death by any cause or censored at the last date of confirmed survival. Progression-free survival (PFS) was defined as the period from the date of treatment initiation to the date of confirmed progression, recurrence, or death by any cause or censor at the last date of confirmed survival. Tumor response was also evaluated in patients with target lesions according to the RECIST ver1.1. The median OS and PFS were estimated using the Kaplan–Meier and the reverse Kaplan–Meier methods for calculating the median follow-up period. All data were analyzed using EZR version 4.2.2. (The R Foundation for Statistical Computing, Vienna, Austria).

## Results

### Patient characteristics

A total of 30 patients were subjected to this study. Patients’ characteristics are shown in Table 1. Six patients were treated as 1L and 24 as ≥ 2L settings. Median age was 70 years (range 51–80) in 1L and 63 years (range 36–77) in ≥ 2L groups. All 6 patients were male in 1L and 18 in ≥ 2L groups. ECOG PS of 0/1 was 2 (33%)/4 (67%) in the 1L and 14 (58%) / 10 (42%) in ≥ 2L groups, respectively.

**Table 1:** Patient characteristics

	All patients, n = 30 (%)	1L Group, n = 6 (%)	≥ 2L Group, n = 24 (%)
Age, median (range), years	63.5 (36–80)	70 (51–80)	63 (36–77)
Sex, n (%)			
Male	24 (80.0)	6 (100.0)	18 (75.0)
Female	6 (20.0)	0	6 (25.0)
Disease status, n			
Advanced	4 (13.3)	1 (16.7)	2 (8.3)
Recurrence	26 (86.7)	5 (83.3)	22 (91.7)
ECOG performance status, n			
0	16 (53.3)	2 (33.3)	14 (58.3)
1 ≤	14 (46.7)	4 (66.7)	10 (41.7)
Smoking status, n			
Current or former smoker	28 (93.3)	6 (100)	22 (91.7)
Never smoked	2 (6.7)	0	2 (8.3)
PD-L1			
TPS ≥ 1% and CPS ≥ 10	7 (23.3)	2 (33.3)	5 (20.8)
TPS ≥ 1% and CPS unknown	9 (30.0)	1 (16.7)	8 (33.3)
TPS < 1% and CPS ≥ 10	2 (6.7)	1 (13.3)	1 (4.2)
TPS < 1% and CPS unknown	7 (23.3)	0	7 (29.2)
TPS < 1% and CPS < 10	4 (13.3)	1 (13.3)	3 (12.5)
Unknown	1 (3.3)	1 (16.7)	0
Base line neutrophil/lymphocyte ratio, n			
4 ≤	14 (46.7)	0	14 (58.3)
4 >	16 (53.3)	6 (100)	10 (41.7)
Measurable lesion, n			
Yes	26 (86.7)	6 (100)	20 (83.3)
No	4 (13.3)	0	4 (6.7)
Number of organs with metastases, n			
1	13 (43.3)	2 (33.3)	11 (45.8)
2	15 (50.0)	4 (66.7)	11 (45.8)
3	2 (6.7)	0	2 (8.3)
Metastases site*, n			
Lymph node	29 (96.7)	6 (100,0)	23 (95.8)
Lung	6 (20.0)	1 (13.3)	5 (20.8)
Liver	4 (13.3)	3 (50.0)	1 (4.2)
Pleural	3 (10.0)	0	3 (12.5)
Bone	3 (10.0)	0	3 (12.5)
Others	3 (10.0)	0	3 (12.5)
Prior therapy, n			
Surgery	13 (43.3)	2 (33.3)	11 (45.8)
Radiotherapy	20 (66.7)	2 (33.3)	18 (75.0)
Systemic anticancer therapy	29 (96.7)	5 (83.3)	24 (100)
5-FU	29 (96.7)	5 (83.3)	24 (100)
Cisplatin	27 (90.0)	4 (66.7)	23 (95.8)
Docetaxel	24 (80.0)	2 (33.3)	22 (91.7)
Oxaliplatin	6 (20.0)	2 (33.3)	4 (16.7)
Paclitaxel	2 (6.7)	0	2 (8.3)
Nivolumab	3 (10.0)	0	3 (12.5)
Treatment cycle, median (range)	2 (1–10)	3.5 (1–6)	2 (1–10)
Median follow-up time (months, 95%CI)	9.0 (6.5–11.6)	9.5 (6.3-NA)	9.0 (6.4–10.5)

*ECOG* eastern cooperative oncology group, *PD-L1* programmed cell death Ligand 1, *TPS* tumor proportion score, *CPS* combined positive score, *NA* not applicable

Metastases site*: includes duplicate cases with multiple metastatic organs in the same case

### Safety

Patients who experienced any grade and grade ≥ 3 irAEs were 3 (50%) and 1 (16.7%) in the 1L and 9 (37.5%) and 4 (16.7%) in ≥ 2L groups, respectively. Details of irAEs and their frequency in the overall population, 1L, and ≥ 2L groups are listed in Table 2. Grade ≥ 3 irAEs was observed in 5 patients; aspartate aminotransaminase increased (n = 2, 6.7%), alanine aminotransaminase increased (n = 2, 6.7%), adrenal insufficiency (n = 1, 3.3%), pneumonitis (n = 1, 3.3%), myositis (n = 1, 3.3%), cytokine release syndrome (n = 1, 3.3%), and Stevens-Johnson syndrome (n = 1, 3.3%), respectively. Among 12 patients who experienced any irAEs, 8 required systemic steroid therapy; low dose prednisolone (PSL): < 1 mg/kg in 1, PSL 0.5 mg/kg in 2, high-dose steroid therapy (PSL, 1 mg/kg) in 3, steroid pulse therapy (methylprednisolone, 1 g/body, day1-3) in 2, respectively. Four patients were resistant to initial steroid therapy: 1 patient was treated with an increased steroid dose (PSL 0.5 mg/kg to 1 mg/kg), 1 with a retry of steroid pulse therapy, 1 with additional mycophenolate mofetil (MMF), and 1 with steroid pulse therapy plus MMF, respectively. As a result, all 8 patients recovered by the above immune-suppression therapy, and no treatment-related death was observed. One patient could stop the steroid therapy and restart Nivo alone. Details of the clinical courses of the 8 patients treated with steroid therapy are described in Table 3.

**Table 2:** Immune related adverse events. (A) All patients, (B) 1L group, (C) ≥ 2L group

(A) All patients						
	All patients, n = 30 (%)					
	Any grade	Grade1	Grade2	Grade3	Grade4	Grade ≥ 3
Any events*	12 (40.0)	5	–	–	–	5 (16.7)
Rash	8 (26.6)	5 (10.0)	3 (10.0)	0	0	0
AST elevation	4 (13.3)	0	2 (6.7)	1 (3.3)	1 (3.3)	2 (6.7)
ALT elevation	4 (13.3)	0	2 (6.7)	1 (3.3)	1 (3.3)	2 (6.7)
Enterocolitis	1 (3.3)	0	1 (3.3)	0	0	1 (3.3)
Hypothyroidism	2 (6.7)	0	2 (6.7)	0	0	1 (3.3)
Adrenal insufficiency	2 (6.7)	0	1 (3.3)	1 (3.3)	0	1 (3.3)
Pneumonitis	1 (3.3)	0	0	1 (3.3)	0	1 (3.3)
Myositis	1 (3.3)	0	0	1 (3.3)	0	1 (3.3)
CRS	1 (3.3)	0	0	0	1 (3.3)	1 (3.3)
SjS	1 (3.3)	0	0	1 (3.3)	0	1 (3.3)

(B) 1L group						
	1L group, n = 6 (%)					
	Any grade	Grade1	Grade2	Grade3	Grade4	Grade ≥ 3
Any events*	3 (50.0)	–	–	–	–	1 (16.7)
Rash	2 (33.3)	2 (33.3)	0	0	0	0
AST elevation	1 (16.7)	0	1 (16.7)	0	0	0
ALT elevation	1 (16.7)	0	1 (16.7)	0	0	0
Enterocolitis	0	0	0	0	0	0
Hypothyroidism	1 (16.7)	0	1 (16.7)	0	0	0
Adrenal insufficiency	2 (33.3)	0	1 (16.7)	1 (16.7)	0	1 (16.7)
Pneumonitis	0	0	0	0	0	0
Myositis	0	0	0	0	0	0
CRS	1 (16.7)	0	0	0	1 (16.7)	1 (16.7)
SjS	0	0	0	0	0	0

(C) 2nd or later line						
	≥ 2L group, n = 24 (%)					
	Any grade	Grade1	Grade2	Grade3	Grade4	Grade ≥ 3
Any events*	9 (37.5)	–	–	–	–	4 (16.7)
Rash	6 (25.0)	3 (12.5)	3 (12.5)	0	0	0
AST elevation	3 (12.5)	0	1 (4.2)	1 (4.2)	1 (4.2)	2 (8.3)
ALT elevation	3 (12.5)	0	1 (4.2)	1 (4.2)	1 (4.2)	2 (8.3)
Enterocolitis	1 (4.2)	0	1 (4.2)	0	0	0
Hypothyroidism	1 (4.2)	0	1 (4.2)	0	0	0
Adrenal insufficiency	0	0	0	0	0	0
Pneumonitis	1 (4.2)	0	0	1 (4.2)	0	1 (4.2)
Myositis	1 (4.2)	0	0	1 (4.2)	0	1 (4.2)
CRS	0	0	0	0	0	0
SjS	1 (4.2)	0	0	1 (4.2)	0	1 (4.2)

**Table 3:** irAEs that needed immunosuppressive therapy

No.	Line	Event	Gr	Initial steroid	Response*^a^	Additional treatment	Response*^b^	Relapse
1	3rd	Enterocolitis	2	PSL 10 mg/body	Yes	–	–	No
2	2nd	AST, ALT Elevation	2	PSL 0.5 mg/kg	Yes	–	–	No
3	2nd	Pneumonitis	3	mPSL Pulse	Yes	–	–	No
4	2nd	SjS Myositis	3	PSL 1 mg/kg	Yes	–	–	No
			3					
5	1st	AST, ALT Elevation	2	PSL 0.5 mg/kg	No	PSL 1 mg/kg	Yes	No
6	1st	CRS	4	mPSL Pulse	No	Re pulse	Yes	No
7	2nd	AST, ALT Elevation	4	PSL 1 mg/kg	No	MMF	Yes	No
8	2nd	AST, ALT Elevation	3	PSL 1 mg/kg	No	mPSL pulse plus MMF	Yes	No

*Gr* grade, *AST* aspartate aminotransaminase, *ALT* alanine aminotransaminase, *CRS* cytokine release syndrome, *SjS* Stevens-Johnson syndrome, *PSL* prednisolone, *mPSL* methylprednisolone, *MMF* mycophenolate mofetil

^*^^a^Response to initial treatment

^*b^Response to additional treatment, mPSL pulse: mPSL 1 g/day administered day 1–3

### Efficacy

Regarding the tumor response, 6 patients in 1L and 20 in ≥ 2L groups with target lesions were evaluated. In the 1L group, 4 patients achieved partial response (PR) and stable disease (SD) in 1, resulting in the best overall response (BOR) of 66.7% (95%CI; 29.6–90.7%) and disease control rate (DCR) of 83.3% (95%CI; 41.8–98.8%). Three patients had TPS ≥ 1% in the 1L group, and 2 patients achieved PR and SD in 1. In the ≥ 2L group, 7 patients achieved PR and SD in 2, resulting in BOR of 36.8% (95%CI; 19.0–59.0%) and DCR of 47.4% (95%CI; 27.3–68.3%). Fourteen patients had TPS ≥ 1% in the ≥ 2L group, and 6 patients achieved PR, SD in 3, and PD in 2. Tumor response is shown in Table 4.

**Table 4:** Best response

Best response	1L Group	≥ 2l Group
	n = 6* (%)	n = 20* (%)
CR	0	0
PR	4 (66.7)	7 (36.8)
SD	1 (16.7)	2 (10.5)
PD	1 (16.7)	10 (52.6)
NE	0	1 (5.0)
ORR	66.7%	36.8%
DCR	83.3%	47.3%

*CR* complete response, *PR* partial response, *SD* stable disease, *PD* progression disease, *NE* not evaluated, *ORR* overall response rate, *DCR* disease control rate

^*^Response was evaluated in 6 and 20 patients with target lesions

**Fig. 1:**
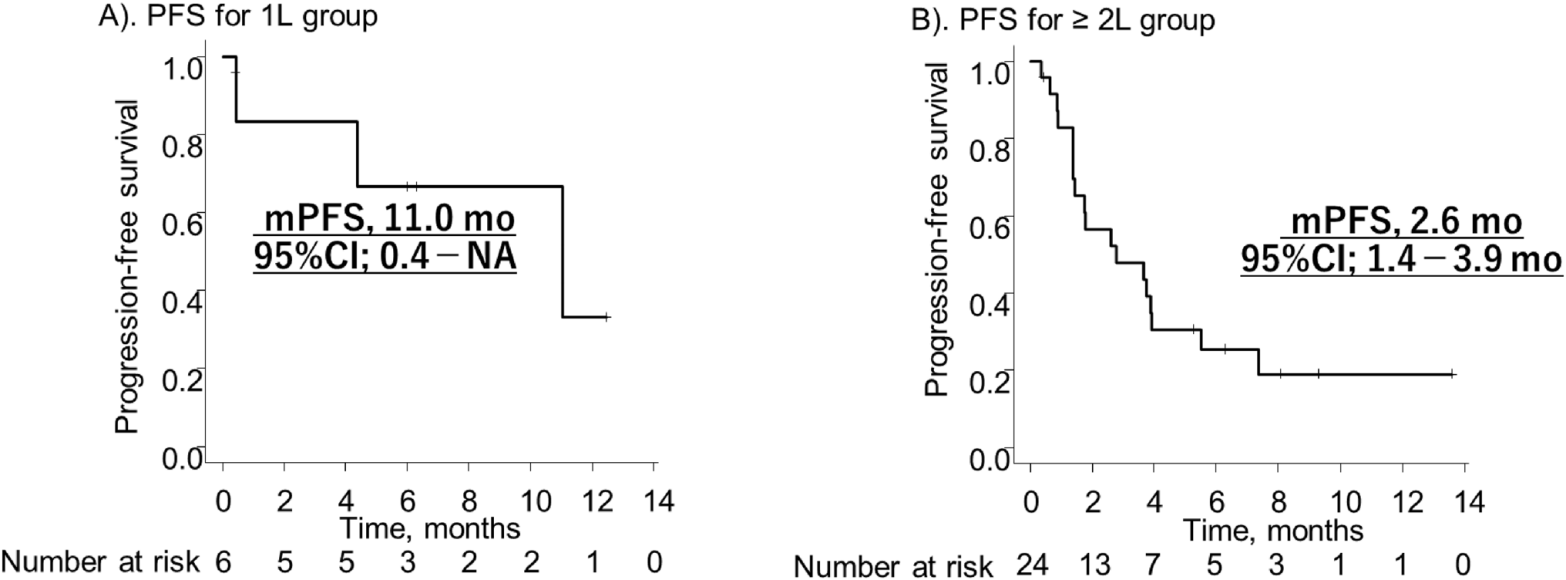
Kaplan-Meier of progression-free survival **A** 1L group. **B** ≥ 2L group. *mPFS* median progression-free survival, *mo* months, *CI* confidenece interval, *NA* not applicable

**Fig. 2:**
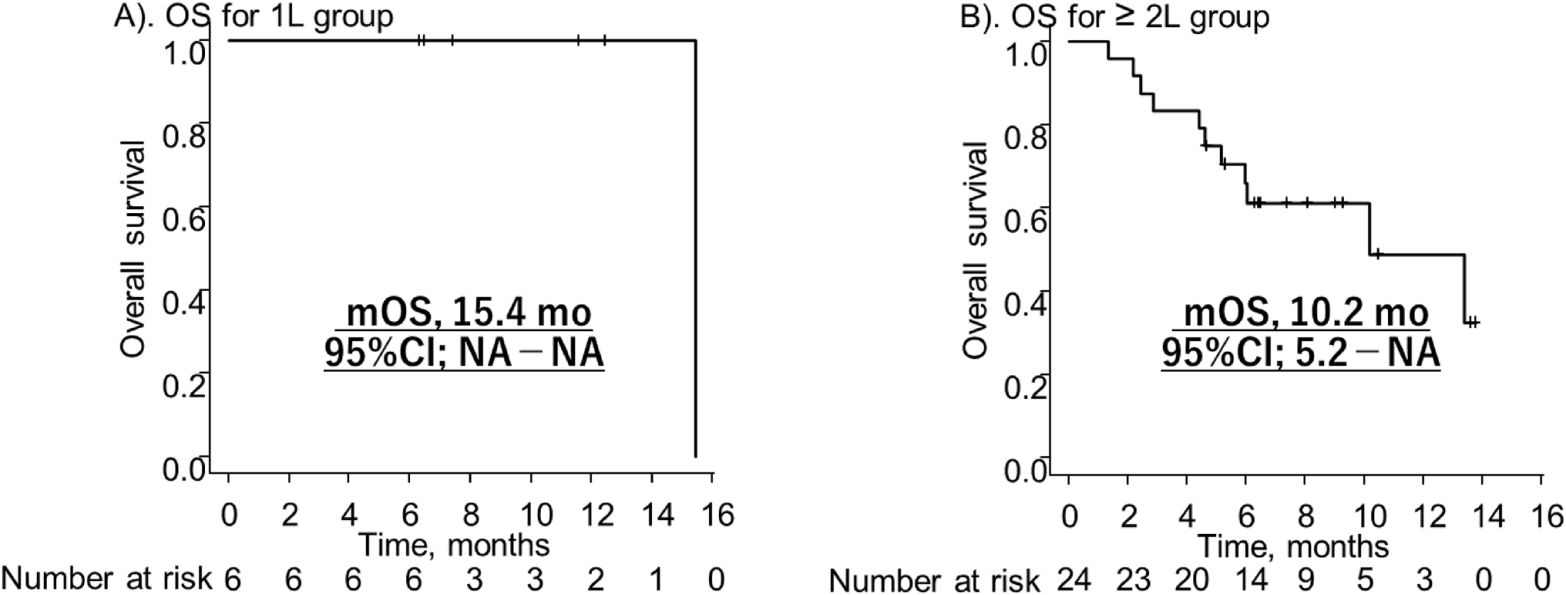
Kaplan-Meier of overall survival **A** 1L group, **B** ≥ 2L group. *mOS* median overall survival, *mo* months, *CI* confidence interval, *NA* not applicable

The median follow-up period of all patients was 9.0 months (95%CI; 6.5–11.6 months), 9.5 months (95%CI; 6.3–not applicable [NA] months) in the 1L group, and 9.0 months (95%CI; 6.4–10.5 months) in the ≥ 2L group. The median PFS and OS were 10.2 (95%CI; 5.2–NA and 15.4 months (95%CI; NA–NA) in 1L group (Fig. 1A, B). and 2.6 (95%CI; 1.4–3.9) and 11.0 months (95%CI; 0.4–NA) in ≥ 2L group (Fig. 2A, B), respectively. In the TPS ≥ 1%, the median PFS and OS were 11.0 (95%CI; 4.4–NA) and 12.5 months (95%CI; 11.6–NA) in 1L group and 3.9 (95%CI; 1.8–NA) and 9.0 months (95%CI; 6.3–10.5) in the ≥ 2L group.

## Discussion

In our study, the incidence of irAEs of any grade and grade ≥ 3 was 40.0% and 16.7%, respectively, aligning with the trends observed in the CM648 trial, where any grade and grade ≥ 3 TRAEs were reported at 80% and 32% for Nivo + Ipi therapy [5]. Notably, the frequency of irAEs in this real-world analysis appears slightly lower than that reported in CM648, potentially reflecting a small population and short-term follow-up time, and our study included a broader range of patients, including those treated in ≥ 2L settings, which may have influenced the observed irAE rates. The RAMONA trial is a multicenter open-label phase II trial assessing Nivo + Ipi and Nivo alone in elderly patients (≥ 65 years) in the 2L setting [7]. The occurrence of irAEs in the RAMOMA trial (56.8% any grade, 18.2% grade ≥ 3) and the frequency of grade ≥ 3 toxicity were similar in the RAMOMA trial and the present study (37.5% any grade, 16.7% grade ≥ 3). Interestingly, the occurrence of irAEs in the 1L group (50% any grade, 16.7% grade ≥ 3) vs. the ≥ 2L group (37.5% any grade, 16.7% grade ≥ 3) did not show a significant difference in the present trial.

The types of grade ≥ 3 irAEs observed in our study included hepatic dysfunction (aspartate aminotransaminase increased, alanine aminotransaminase increased), adrenal insufficiency, pneumonitis, myositis, cytokine release syndrome, and Stevens-Johnson syndrome. Although their relative frequencies differed, these toxicities are consistent with those reported in the CM648 trial [5].

Several guidelines for managing irAEs include the American Society of Clinical Oncology guidelines [8], National Comprehensive Cancer Network guidelines [9], the Society for Immunotherapy of Cancer [10], and the European Society for Medical Oncology consensus guidelines [11]. Our data highlight the critical importance of timely and appropriate management of irAEs. Among the 12 patients who experienced irAEs, 8 required systemic steroid therapy, and 4 were resistant to initial treatment. Notably, the use of MMF in steroid-refractory cases was effective, allowing all patients to recover without treatment-related mortality. Previous report showed that early combination with MMF in addition to systemic steroids can lead to a more rapid improvement of immuno-related hepatitis compared to late combination with MMF, consequently reducing the required systemic steroid dosage [12]. This report indicated that early use of MMF may be more effective than late use. Early initiation of MMF in steroid-refractory cases may also improve outcomes by reducing the risk of prolonged irAE-related morbidity. Given the potential for severe and refractory irAEs, we should maintain a low threshold for introducing MMF or other immunosuppressive agents when initial steroid therapy fails. This strategy may help optimize patient recovery and minimize treatment interruptions.

The overall efficacy of Nivo + Ipi in the present study showed promising results, with a median OS of 15.4 months and PFS of 10.2 months in the 1L group and 12.5 months and 11.0 months in the 1L group with TPS ≥ 1%. Compared to the CM648 trial regarding these results, the TPS of ≥ 1% was slightly lower regarding OS, and the PFS was much longer. However, caution is needed with these results due to small sample size and short follow-up duration. Additionally, although this was real-world data, the patient backgrounds at our hospital were not significantly different from those in clinical trials. Additionally, aggressive cases were avoided based on information of CM648, which may have influenced these results.

In the ≥ 2L group, the median OS of 11.0 months and PFS of 2.6 months were notably lower, reflecting the challenges of treating heavily pretreated populations. Poor efficacy of the ≥ 2L Nivo + Ipi compared with the 1L therapy has also been reported in other cancer types, with a report of real-world data in malignant pleural mesothelioma showing a PFS of 6.5 months for the 1L therapy compared with 2.8 months the ≥ 2L therapy [13]. Of the 24 cases, 20 experienced recurrence within 6 months after completing preoperative chemotherapy or definitive chemoradiotherapy, and another 2 cases recurred during adjuvant nivolumab therapy. These recurrences may reflect a disease state less responsive to drug therapy. In the present study, the shorter PFS of Nivo + Ipi in the ≥ 2L group is due to a higher proportion of patients with a neutrocyte/lymphocyte ratio of ≥ 4 and a higher proportion of cases with organ metastases other than lymph node metastases. However, the shorter PFS and prolongation of median OS were shown. This OS prolongation might be mainly because (i) the rate of receiving subsequent therapy and (ii) Nivo + Ipi itself may increase the efficacy of subsequent therapy. First, 6 patients continued the Nivo + Ipi, and 18 patients were discontinued in the ≥ 2L group at the data cut-off. The reason for discontinuation was disease progression (n = 16, 75.0%) and sudden death other than carcinoma (n = 1, 4.0%) and toxicity (n = 1, 4.0%). In the disease progression, 15 patients (83.3%) received subsequent chemotherapy. Second, Nivo + Ipi might affect the efficacy of subsequent therapy. The CheckMate 227 trial showed longer PFS2 in Nivo + Ipi than chemotherapy combined with Nivo, both PD-L1 ≥ 1% (14.8 vs. 12.0 months) or 1% > (14.2 vs. 12.3 months) [14]. On the other hand, as for aESCC, in a retrospective study examining the therapeutic effect of taxanes after Nivo therapy in the Japanese population of the ATTRACTION-3 (AT-3) trial, the response rate and the median PFS in the third-line treatment after Nivo were 29.6% and 4.9 months, respectively [15]. These results were not so different from those of taxanes in the AT-3 trial. In contrast, our previous report showed that the ORR in the previously treated and naive groups with ICIs were 37.5% (12/32) and 13.4% (9/67), respectively, showing a statistically significant difference (p = 0.007). Still, the median PFS was 3.8 months in the previously treated group and 2.8 months in the naive group, respectively, and no statistically significant difference was observed (p = 0.99) [16]. Thus, although Nivo therapy may potentially increase the ORR to subsequent therapy, a prolongation of PFS has not been statistically demonstrated. Regarding Nivo + Ipi, there is insufficient data due to the lack of historical controls, the small number of patients, and the short follow-up period in the present study, which may be controversial. A multicenter study is currently being considered, and future results are warranted.

This study has several limitations. First, this is a retrospective study with a relatively small sample size. Second, follow-up time is short, and longer follow-up is needed to evaluate the efficacy. Third, there is a selection bias in the use of Nivo + Ipi. Fourth, the point that patients from different treatment lines were assessed together with regard to the ≥ 2L.

## Conclusion

This study provides valuable insights into the real-world safety and efficacy of Nivo + Ipi in advanced ESCC compared to the results of the CM648 trial, and Nivo + Ipi showed promising efficacy as the ≥ 2L treatment. While the toxicity profile was manageable with appropriate interventions, including early use of MMF, further studies are needed to refine patient selection criteria and management strategies.

## Data Availability

The data that support the findings of this study are available from the corresponding author upon reasonable request.
